# Program for Integral Assistance to Women's Health, 40 years of history: interview with Ana Maria Costa

**DOI:** 10.1590/S0104-59702024000100029

**Published:** 2024-06-17

**Authors:** Ana Maria Costa, Claudia Bonan, Andreza Pereira Rodrigues

**Affiliations:** i Professora, Pós-graduação em Saúde Coletiva/Escola Superior de Ciências da Saúde/Universidade do Distrito Federal. Brasília – DF – Brasil dotorana@gmail.com; ii Professora, pesquisadora, Instituto Nacional de Saúde da Mulher, da Criança e do Adolescente Fernandes Figueira/Fiocruz. Rio de Janeiro – RJ – Brasil bonanclaudia@gmail.com; iii Professora, Escola de Enfermagem Anna Nery/Universidade Federal do Rio de Janeiro. Rio de Janeiro – RJ – Brasil andrezaenfermeira@gmail.com

**Keywords:** Women’s health, Reproductive health, Comprehensive health care, Public policy, Health policy, Saúde das mulheres, Saúde reprodutiva, Assistência integral à saúde, Política pública, Política de saúde

## Abstract

The interview marks the 40th anniversary of the Programa de Assistência Integral à Saúde da Mulher (Program for Integral Assistance to Women’s Health), and aims to revisit the history of this innovative health policy, the context in which it was created and the generation that took it forward, from the narrative of a key person, Ana Maria Costa, who played a leading role in the process of its creation, from conception to the elaboration of its final text. Launched in 1983, the policy was a pioneer in proposing and incorporating the principles of universality, equity and integrality, which would be the foundations of the Sistema Único de Saúde, and introducing the perspective of women’s reproductive rights.

## Resumo

A entrevista marca os 40 anos do Programa de Assistência Integral à Saúde da Mulher e tem como objetivo revisitar a história dessa política de saúde inovadora, do contexto em que foi criada e da geração que a levou adiante, a partir da narrativa de uma pessoa-chave, Ana Maria Costa, que protagonizou o processo de sua criação, desde a concepção até a elaboração de seu texto final. Lançada em 1983, a política foi pioneira em propor e incorporar os princípios de universalidade, equidade e integralidade, que seriam os fundamentos do Sistema Único de Saúde, e introduzir a perspectiva dos direitos reprodutivos das mulheres.

Ana Maria Costa, médica sanitarista, militante da reforma da saúde, ativista feminista, gestora de saúde, pesquisadora e professora universitária, tem uma carreira profissional e política que se confunde com a própria história do Programa de Assistência Integral à Saúde da Mulher. Conhecida como Paism, essa política foi lançada em 1983, ainda sob um regime ditatorial civil-militar, alguns anos antes de eventos como a oitava Conferência Nacional de Saúde (1986) e a Assembleia Nacional Constituinte (1986-1988), que consagraram a universalidade, a equidade e a integralidade como princípios fundamentais do Sistema Único de Saúde (SUS) (Osis, 1998; Costa, 2012; Paiva, Teixeira, 2014).

Como técnica da Divisão Nacional de Saúde Materno-infantil do Ministério da Saúde, do final dos anos 1970 até sua extinção, no início da década de 1980, Ana foi testemunha e partícipe das controvérsias que envolveram a questão do “planejamento familiar” e as tentativas de introdução de um programa estatal de controle demográfico no Brasil na segunda metade do século XX. E, ainda mais relevante, ela foi pessoa-chave na concepção e elaboração de uma política de saúde inovadora, que ofereceria uma alternativa progressista a essas controvérsias, fundamentada nos princípios da integralidade na atenção à saúde, da autonomia e dos direitos reprodutivos das mulheres, o Paism. É a história dessa política, do contexto em que ela foi criada, das disputas que estavam envolvidas e da geração que a levou adiante que Ana nos conta nesta entrevista, realizada em maio de 2023, ano em que o Paism chegou aos seus 40 anos.

**Figura 1:**
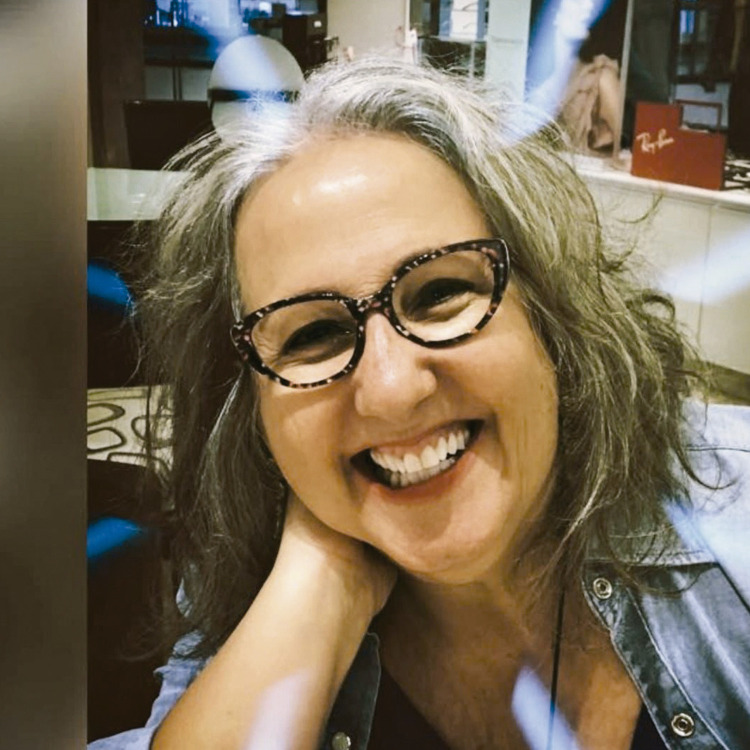
: Ana Maria Costa, 2017 (acervo pessoal)

*Claudia Bonan: Ana, obrigada por nos conceder a entrevista, neste ano em que celebramos os 40 anos do lançamento do Paism. Conte um pouco sobre você, como chegou à saúde pública, ao feminismo, ao Ministério da Saúde.*

Eu fiz medicina na universidade de Darcy Ribeiro, a Universidade de Brasília (UnB), onde o projeto pedagógico curricular era alinhado ao conceito de universidade como lugar de conhecimento universal, das grandes disciplinas de ciências. O vanguardismo do curso de medicina da UnB só tinha similar no curso experimental de medicina da Universidade de São Paulo. Para vocês terem uma ideia, a Faculdade de Medicina tinha uma regional-escola, numa cidade-satélite, gerida pela universidade. Ali havia uma rede de serviços na qual os alunos transitavam e que juntava formação, docência, assistência e pesquisa. Foi um lugar de inovação das ideias e práticas. Buscando garantir melhores níveis de saúde surgem estratégias como “busca ativa”, “agente de saúde”, “consultas coletivas”, entre outras.

Nossa formação foi impregnada de inquietações coletivistas. Cada aluno recebia um território e tinha que dar conta do que acontecia ali, com a população. Nós estávamos tanto no hospital quanto nos postos de saúde, enfim, no território. De fato, era uma formação muito integrada e integradora.

*Andreza Rodrigues: Então, foi nessas universidades públicas – de Brasília, São Paulo e outras – que se gestaram conceitos centrais do SUS, como integralidade, e se formaram as primeiras gerações do movimento de reforma sanitária?*

Posso afirmar que a UnB formou os grandes teóricos e ativistas do movimento da reforma sanitária, como Eleutério Rodrigues Neto, Gastão Wagner, Luiz Cecílio, Ricardo Lafetá, só para citar algumas das pessoas originadas desse modelo de formação fundado no pensamento crítico e coletivista, o que faz grande diferença. Quem se forma nesse contexto nunca mais é o mesmo. Não se pode equiparar com quem tem uma formação intervencionista, voltada para o hospital e a alta tecnologia. Qualquer que seja a especialidade futura do aluno formado na perspectiva de uma compreensão da complexidade e multicausalidade do processo saúde/doença, o raciocínio complexo sobre saúde fará dele um profissional diferenciado. Nós fomos formados dentro de um conceito de determinação social do processo saúde/doença, de saúde como algo que não se faz apenas com assistência médica, mas saúde como o produto de uma acumulação social, de qualidade de vida da população.

*Claudia Bonan: Então, você bebeu na fonte do projeto de uma “universidade-semente”, como dizia Darcy Ribeiro. Uma universidade capaz de gerar pensamento crítico, de contribuir para o desenvolvimento social e cultural do país, de participar do enfrentamento das iniquidades e exclusões.*

Eu me formei em 1977. Foi a última turma que bebeu dessa fonte, do que tinha restado dela. Eu bebi das últimas gotas da fonte, pois muitos professores depois foram banidos da universidade, exilados. Foram momentos muito difíceis, mas ainda houve alguma resiliência, mantida exatamente pela nossa inserção no território. Era o território que ainda nos segurava, a Unidade Integrada de Saúde de Sobradinho, essa regional-escola. Nós praticamente morávamos ali, nos sentíamos pertencentes ao território e tínhamos um grande compromisso com a população. Ao lado dessa formação privilegiada, o movimento estudantil da UnB foi valente na luta contra a ditadura; e foi também lugar de formação de pensamento político. Então, foi essa a universidade que eu vivi.

*Claudia Bonan: Como a semente do movimento sanitário foi germinando e se tornou força política e intelectual importante, no Brasil e América Latina, além de foco de resistência à ditadura?*

Naquela época, apesar da ditadura, tinha uma onda ascendente de pensamento crítico em saúde, a base da saúde coletiva, com a influência do pensamento latino-americano sobre a determinação social da saúde, a tese do Sérgio Arouca, a produção seminal de Cecília Donnangelo... Então, eles trouxeram questionamentos substantivos ao pensamento médico sobre saúde, hegemônico no Brasil.

O estado de São Paulo, em 1969, tinha criado uma carreira de sanitarista, e ali estavam Gastão Wagner, Luiz Cecílio e outros. Também se criou na Universidade de São Paulo um curso curto de saúde pública, que tinha a intenção de prover pessoal para essa precursora carreira de sanitarista. A Escola Nacional de Saúde Pública também já oferecia formação para sanitarista.

*Andreza Rodrigues: Você fez esse curso?*

Fui fazer aquele curso curto e entrei na carreira de sanitarista em São Paulo. Simultaneamente, fui convidada a ir para o ministério, então voltei para Brasília. Entrei como contratada especial, em 1977, na Divisão de Saúde Materno-infantil, por algum daqueles mecanismos chamados de “cargo de confiança”.

*Andreza Rodrigues: Como era a Divisão de Saúde Materno-infantil do ministério nessa época?*

O ministro era Paulo de Almeida Machado, e o chefe da Saúde Materno-infantil era Ciro Coimbra de Resende. A área de Divisão de Saúde Materno-infantil era muito bizarra! Tinha umas trinta pessoas. Eu era uma menina de 27-28 anos, chegando ali, toda “riponga”. A maioria era de enfermeiras, e também havia médicos. Elas eram todas muito formais. Tinham uma idade mais avançada, mas não era só a idade, era a formalidade de senhoras. Um ministério velho, do ponto de vista de ideias antigas, verticais e normativas. Com uma centralização excessiva. Isso se modificou depois, com o SUS, mas essa cultura pode ser identificada ainda hoje!

*Andreza Rodrigues: Na prática, como era essa questão da verticalização e centralização das ações de saúde?*

Para você ter ideia, eu tinha a função de cuidar da área materno-infantil da região Norte. Eu planejava o que cada cidade da região deveria oferecer, ou seja, quantas consultas pré-natal, quantos acompanhamentos de crescimento e desenvolvimento infantil, quantos partos a cidade deveria assistir etc. Feito o planejamento, era mandado à cidade aquele pacote das metas que eles tinham que perseguir, atingir. Era uma coisa absurda! Aplicavam-se os mesmos parâmetros demográficos, epidemiológicos e de cobertura para todas as cidades do país. Os critérios não deveriam ser homogêneos, já que o Brasil é tão diverso e desigual! Algumas enfermeiras eram especializadas em calcular até o número de sabonetes, o material de esterilização... Enfim, tudo muito centralizado.

Feito o pacote, cabia acompanhar para ver se o que foi planejado foi realmente feito. Mas eram postos de saúde de prefeituras muito limitadas, não tinham atribuições plenas de atenção primária, não tinha nenhuma política maior que pudesse dar conta daquilo. Na Divisão Materno-infantil, também tínhamos a burocracia de autorizar material que entrava no Brasil, para atendimento da área. Uma vez chegou um pedido de parecer para eu dar sobre a entrada de dispositivo intrauterino (DIU) no país.

*Andreza Rodrigues: Era para algum programa de planejamento familiar?*

O assunto do planejamento familiar no ministério foi assim: o Programa de Saúde Materno-infantil, de 1974, foi o primeiro a falar do tema, mas preconizava somente atividades de aconselhamento e orientação para espaçamento das gestações. Em 1977, o ministério mobilizou um conjunto de médicos acadêmicos para discutir o assunto do planejamento familiar, e foi criado um programa muito criticado, de controle populacional, o Programa de Prevenção da Gravidez de Alto Risco, conhecido como PPGAR, que propunha oferecer métodos às mulheres de “alto risco”. O crivo para demarcar os fatores de risco eram basicamente critérios raciais e sociais. Vaticinavam o destino reprodutivo do risco das mulheres negras e pobres. O que teve foi mais uma investida de controle da natalidade, de cima para baixo, como já vinha sendo feito desde os anos 1960, com entidades como a Bemfam.^1^

Houve reação do movimento sanitário, das feministas e de setores da esquerda, que acusaram o governo de promover o controle demográfico. Até no *Pasquim* apareceu a Graúna denunciando a Bemfam, que incidia nos municípios para promover uma barbárie – uma assistência nada saudável ou respeitosa para as mulheres –, que era a distribuição indiscriminada de contraceptivos hormonais. Havia também uma grande interiorização das ações de outra entidade privada de planejamento familiar, o CPAIMC,^2^ do Hospital Escola São Francisco de Assis, ligado à Universidade Federal do Rio de Janeiro, dirigido pelo Hélio Aguinaga.

O Aguinaga chegava lá no ministério para fazer tratativas a fim de liberar na Alfândega os equipamentos que ele trazia do exterior para fazer o trabalho pouco ético dele, que era exatamente a esterilização cirúrgica. Foi ele quem introduziu no Brasil a tecnologia da esterilização feminina pela via laparoscópica. Ele também se ocupava da implantação de DIU. Aliás, tenho que fazer justiça, ele importava diafragmas também.

*Claudia Bonan: Como você se aproximou das discussões sobre planejamento familiar e controle demográfico naqueles anos?*

Meu incômodo já existia antes do ministério. Foi exatamente nesse assunto que me meti lá no curso de sanitarista de São Paulo. Eu tinha vários companheiros e companheiras lá que eram altamente informados sobre esse tema. Participei de debates sobre essa questão na Associação Paulista de Sanitaristas. Era questão nacional naquele tempo. Nós não podemos esquecer que a direita atribuía a pobreza no Brasil ao avanço do crescimento demográfico. Diziam “somos pobres e desiguais porque nascemos muito”. Havia essa máxima.

*Claudia Bonan: Essa era também a visão do regime militar quando você estava no ministério?*

Sim, o ministro da Justiça da época, o do João Figueiredo, no seu discurso de abertura de 1982, defendeu que não haveria desenvolvimento no Brasil se não houvesse planejamento familiar. Na verdade, ele queria dizer, se não houvesse redução e controle do crescimento das populações pobres. Eu estava no ministério nesse momento, e foi isso que deu o mote para a gente apresentar ao país uma alternativa, uma política de saúde integral das mulheres.

*Andreza Rodrigues: Nesse momento já estava pronta a proposta do Paism?*

Estava a caminho. Nos pródomos de 1977 e 1978, quando começa a importação dos equipamentos para esterilização cirúrgica e métodos contraceptivos, eu começo a descobrir uma possibilidade de intervir, como técnica, naqueles pareceres que eram solicitados para liberação desses produtos. Um dia, um médico do ministério, de formação cristã, doutor Orlando, me procura para fazer uma aliança, porque sabia que eu estava dando esses pareceres. Ele achava que era uma violência cristã o planejamento familiar. Eu estava preocupada com o outro lado, com a violação dos direitos e a saúde das mulheres, mas construímos ali uma aliança, e ele passou a drenar todos os processos para mim. Eu tinha uma legitimidade na área para fazer aquilo, e ele passou a dar um jeito de mandar tudo para mim.

*Andreza Rodrigues: Aliança entre perspectivas tão diferentes, você com olhar feminista, preocupada com os direitos das mulheres, e o médico católico que, por motivos religiosos, recusava os métodos contraceptivos.*

Os motivos eram completamente diferentes, mas eram alianças táticas, como acontece em contextos políticos muito restritos. Eu não tinha muitas alternativas, não tinha uma rede ali dentro com a qual me aliar para defender os direitos das mulheres. Eu me lembro que nossa conversa era quase como uma coisa de aparelho, ele falando comigo discretamente, como nos velhos aparelhos políticos da clandestinidade.

*Claudia Bonan: Você então era a única pessoa, nessa época, com um olhar mais feminista, de direitos reprodutivos, na Divisão Materno-infantil?*

Na mesma área, veio trabalhar comigo uma socióloga muito bem preparada: Graça Ohana. Uma pessoa muito competente e discreta, não era uma pessoa de briga como eu, de enfrentamento, mas me provia de informações e argumentos. Então a gente fez uma parceria profícua, porque ela gostava muito de informação, de organizar dados. Ela ficou com essa atribuição, e eu ia para o *front*, fazer o embate. Assim fomos crescendo no ministério, como uma dupla que tinha uma franca oposição à forma como esses produtos eram utilizados, porque envolviam riscos para as mulheres.

*Claudia Bonan: Vocês já tinham aliança com o movimento da reforma sanitária?*

Nesses anos, o movimento da reforma sanitária avançava e começava a propor os arranjos que precederam a própria proposta de criação do SUS. Você tinha o Programa de Interiorização de Ações de Saúde e Saneamento, depois vieram Ações Integradas de Saúde, e por aí fomos formando espaços de discussão e avançando no debate. Eram espaços democráticos e progressistas. Uma das estratégias que nós da reforma sanitária tivemos, que foi muito eficaz, foi entrar e influenciar espaços do governo. A gente cunhou uma marca lá dentro.

*Claudia Bonan: Então já havia outros sanitaristas progressistas atuando no ministério?*

Você chegava no ministério, tinha um bando de sanitaristas formados em São Paulo, Rio de Janeiro ou outro lugar. Nós éramos conhecidos como os esquerdistas que trabalhavam, que eram excelentes para trabalhar! [risos]. Eu trabalhava pra caramba, propunha inovações, porque também era uma estratégia construir mudanças por dentro da própria máquina. Desconheço um acordo estratégico do tipo “vamos invadir a máquina!”. Talvez o Partido Comunista Brasileiro o tivesse. Mas identifico uma vontade grande de avançar, porque o projeto do SUS, do direito universal à saúde, já tinha nos contaminado, e nos declarávamos integrantes do Partido Sanitário, um projeto coletivo que se colocava para este grupo novo, dos novos sanitaristas.

*Andreza Rodrigues: E o feminismo, como chegou?*

Um grupo de mulheres que estavam dentro do movimento sanitário; fomos tomando uma consciência que também nos aproximava do pensamento feminista. Começamos a nos nutrir do pensamento feminista na temática do crescimento demográfico, que fazia uma inflexão diferente daquela que a própria esquerda fazia. A diferença, que eu acho, é que acrescentava, ao marco sanitário da ausência de riscos, um grande marco que é o da autonomia das mulheres.

*Claudia Bonan: Essa foi a perspectiva que embasou o Paism também.*

Sim, articular a questão da contracepção e do planejamento familiar às perspectivas da integridade corporal e ausência de riscos à saúde, do direito reprodutivo e da autonomia das mulheres. Esses valores não estavam postos nas propostas que antecederam o Paism. Nos anos 1970, a Igreja propõe essa ideia de “paternidade responsável” ao ministério, e isso aparece nos programas materno-infantis. Queria que a “paternidade responsável” ocupasse o espaço do que era chamado de “planejamento familiar”, e hoje se chama de “planejamento reprodutivo”.

*Andreza Rodrigues: Ana, você falou “a gente estava lá dentro”, se referindo à presença de pessoas do movimento da reforma sanitária no Ministério da Saúde. Fale mais sobre isso.*

Estávamos inseridos no movimento da reforma sanitária e no ministério; não era “eles lá” e “nós aqui”. Lembro que, quando já tínhamos terminado de escrever o texto do Paism, eu estava em um seminário em Campinas, onde discutíamos operacionalização do programa. O Nelsão [Nelson Rodrigues dos Santos, liderança do movimento sanitário] chega para mim, me dá um abraço e diz assim, “companheira, vocês nos superaram, vocês acharam a saída!”. Veja o seguinte: na reforma sanitária, a gente lidava com uma contradição. Nós feministas também acreditávamos que não tinha que ter política de controle demográfico, que isso servia às políticas de hegemonia norte-americana. Porém, nós acreditávamos também que as mulheres tinham o direito de escolher sobre sua reprodução, tinham que ter autonomia reprodutiva, controlar elas mesmas a sua fecundidade e decidir sem correr riscos à saúde.

*Claudia Bonan: Essas entidades como Bemfam e o CPAIMC, que distribuíam contraceptivos hormonais e faziam esterilização, não garantiam nada disso?*

Não! Nós tínhamos notícias de mulheres que eram esterilizadas sem terem sido informadas, nem opinado. Havia mulheres que descobriram depois de muitos anos estarem usando DIU. Essas violências já vinham acontecendo, nós tínhamos notícias, mas o ministério fazia cara de paisagem, porque todas essas ações eram feitas em acordo direto entre a Bemfam e o CPAIMC com os municípios.

*Claudia Bonan: Conte um pouco mais como as feministas chegaram ao debate da política de saúde integral para as mulheres.*

A gente começa a pensar sobre bases teóricas e éticas que pudessem sustentar uma nova abordagem para a questão da contracepção, diferente daquela do controle demográfico. No início, os debates feministas no Brasil eram muito mais denunciativos do que propositivos, porque a gente não tinha propostas reais acerca do problema. A “gente” que eu estou dizendo é o feminismo, que era muito incipiente nos anos 1970.

Quando inicia a transição democrática no Brasil, com a distensão política e a anistia, começam a voltar mulheres exiladas, e o debate feminista se incrementa. A gente começa a ter mais clareza que a questão do planejamento familiar ultrapassa a própria saúde, para entrar em novas dimensões, dimensões de direitos e autonomia para as mulheres.

*Andreza Rodrigues: Isso não era algo que estivesse ali, pairando no movimento da reforma sanitária…*

Não! Nós que colocamos isso ali em debate!

*Claudia Bonan: Fale mais das relações entre o movimento feminista e o sanitarismo nessa discussão do Paism.*

Olha, quando eu escrevi o Paism, o Davi Capistrano me apresenta para o Arouca num boteco em Brasília e diz: “Olha aqui, a Ana Costa, que acabou de dar um salto, escreveu o Paism”. Aí Arouca olhou: “Ana, eu preciso te contar. Me chamaram no Rio de Janeiro para debater esse documento. As feministas do Rio de Janeiro me chamaram para bater nesse documento”. Para você ver que o feminismo no início não estava hegemônico no apoio ao Paism. Aí Arouca conta que “na hora do debate fiz um rasgado elogio que não tinha nada mais inteligente, mais avançado do que esse documento nos últimos anos na saúde do Brasil!”.

Olha só, isso é muito importante. Eu posso dizer que a discussão do Paism estava fora da reforma sanitária? Não! Ao contrário, ela foi gestada ali dentro. Quando você está discutindo universalização do direito à saúde, qualidade do atendimento, conceito ampliado de saúde, discutindo as grandes bases do movimento da reforma sanitária, é isso que nos dá chão para andar com a proposta do Paism, não tenho a menor dúvida! Não era o feminismo que estava numa movimentação por essa política, ele estava muito mais numa movimentação de questionar, e era correto! Era fase denunciativa, e era corretíssima! Tinha que desconfiar mesmo da formulação do Paism, estávamos vivendo uma ditadura.

*Andreza Rodrigues: Apesar de não estarem alinhadas em bloco com o Paism, apesar das suspeitas, houve feministas que contribuíram?*

Olha, nesse momento da formulação do Paism, tinha uma movimentação em São Paulo. Com o governo Franco Montoro, São Paulo teve o seu primeiro conselho dos direitos das mulheres. Tinha à frente Margareth Arilha e Marina Réa. Graça e eu marcamos uma reunião com Marina, que estava na Secretaria de Saúde, conduzindo algo que viria a ser uma política paulista de saúde da mulher. Apresentamos para ela o que nós estávamos formulando, e Marina olhou para mim e disse assim: “Vocês estão muito mais avançadas do que nós!”. Então, pegou a política da Secretaria de Saúde de São Paulo e o que nós tínhamos já avançado e começamos a trabalhar juntas. Não existia SUS, não havia política nacional que orientasse todo o conjunto. Cada estado tinha sua política, mas a gente fez uma aliança e um trabalho conjunto.

*Claudia Bonan: Como a proposta do Paism prospera dentro do ministério, no início dos anos 1980? Foi quando o governo Figueiredo começou a defender mais abertamente o controle demográfico, argumentando que isso era necessário para desenvolver o Brasil, não é?*

Em 1982, Figueiredo faz o discurso que não tem futuro para o Brasil se não tiver uma interrupção do crescimento demográfico e insta o conjunto dos ministérios a dar resposta ao que ele chamou de “escândalo do crescimento demográfico”. Simultaneamente, é criada uma Comissão Parlamentar de Inquérito (CPI) para investigar o aumento populacional. Se a proposta prosperou no ministério, eu acho que todo mérito tem que ser dado a uma pessoa que abriu todas as portas e entendeu todo aquele momento e aquele discurso. Falo de Mozart de Abreu e Lima, que era o secretário executivo do ministro da Saúde Waldyr Arcoverde. Mozart já tinha sabido que havia duas rebeldes ali dentro do ministério, manda me chamar, e diz: “Vamos formular uma política, nessa linha do questionamento, mas com uma outra visão, eu vou bancar vocês. A gente vai se cercar de alguns grupos que vão nos dar respaldo”. Aí chama a Conferência Nacional dos Bispos do Brasil (CNBB), chama um grupo da Universidade Estadual de Campinas (Unicamp), o José Aristodemo Pinotti, que emprestava seu prestígio acadêmico. Pinotti, que era reitor da Unicamp, não podia estar toda hora com a gente, e botou um auxiliar dele lá. Era um jovem professor que sentava comigo, mas de poucas palavras, que chamava Oswaldo. Logo depois a gente foi fazer um curso na Organização Mundial da Saúde, em Genebra. Botaram Oswaldo para ir comigo, e também convidamos a Marina Réa para ir junto, fomos fazer um curso sobre planejamento familiar, dado por um egípcio. A ideia deles era a gente pegar aqueles programas africanos e criar uma tecnologia de gestão do planejamento familiar.

*Andreza Rodrigues: A interlocução com Pinotti, como era?*

Boa, ele era muito suave. Pinotti começa a disseminar que as conversas conosco imprimiam a ele um outro olhar sobre a medicina, porque uma mulher que ele visitava à beira do leito já não era mais um número, tinha um nome, tinha uma história. Porque a gente começou a formular mudanças de habilidades e condutas nessa linha…

*Andreza Rodrigues: Fale mais do papel do Mozart de Abreu e Lima na história do Paism.*

O Mozart era de fato uma pessoa que peitava a proposta do Paism, ouvia os nossos alertas, acatava as nossas referências e, ao mesmo tempo, nos conduzia a ter uma tolerância de negociação. Por exemplo, com dom Luciano Mendes, da CNBB. Eu não me esqueço do Mozart me chamar às seis horas da tarde e dizer: “Hoje você se prepara, porque nós vamos receber aqui dom Luciano Mendes”. Dom Luciano chegava pelas sete e meia no ministério e saía duas horas da manhã. Não era muita gente não, nós éramos três ou quatro. Dom Luciano fechava o olho e pensava, pensava, e aí falava. O que é que dom Luciano queria? Tentar uma conciliação, porque ele era um homem muito sensato. Ele sabia que já não podia segurar tudo na mão da Igreja.

*Claudia Bonan: E vocês apresentavam o documento que estava em desenvolvimento?*

Não. A gente discutia pontos muito específicos da questão da oferta de métodos. O que ele queria mesmo era que não tivessem métodos “abortivos ou suspeitos de serem abortivos”. A conversa era essa. A ideia da gente e do Mozart não era apresentar documento, não era pegar a bênção dele, não era nada disso!

*Andreza Rodrigues: Como é que vocês o dissuadiam?*

Era dissuadido com muita conversa, muito argumento. Eu e o Mozart conversávamos com dom Luciano sobre o planejamento familiar e aí entremeávamos outras conversas, depois entrávamos de novo no assunto e tal. A conversa chegou a ponto de dom Luciano Mendes me mandar para o Canadá, para uma reunião de um grupo católico chamado Serena, para me convencer sobre a importância, a eficácia e a possibilidade dos métodos comportamentais.

*Andreza Rodrigues: E aí você foi para a reunião do Serena?*

Fui. Era um encontro de casais do mundo inteiro que tinham uma vida de sucesso com os métodos comportamentais. Dom Luciano queria que os postos de saúde tivessem uma sala específica para método comportamental, ele não queria “contaminar” a população católica com a possibilidade de uso de outros métodos. Como se as mulheres católicas não abortassem, não fizessem esterilização cirúrgica...

*Claudia Bonan: Ana, uma pergunta, o movimento negro participou nesse momento?*

Não, nada. O movimento negro participa em outro momento, em que eu também participei. Foi na Comissão Parlamentar Mista de Inquérito (CPMI) da esterilização de mulheres negras, presidida pela Benedita da Silva. Eu fui assessora dessa CPMI, que foi provocada por um documento da Luiza Bairros, do movimento negro da Bahia, em cima daquela terrível propaganda em que Elsimar Coutinho associava morte materna a “defeito de cor”. Isso foi já na época do ministro da Saúde Adib Jatene, em 1990-1991.

*Claudia Bonan: Voltando ao lançamento do Paism, como vocês conseguiram recolocar a questão do planejamento familiar, virando a chave do controle demográfico para a saúde integral das mulheres, na CPI do aumento populacional, de 1983?*

O Mozart era um grande aliado. Tenho que dar esse mérito para ele, tenho a maior admiração! Ele não rompeu o pacto com a gente e com as mulheres, em nenhum momento. Impressionante! Ainda perguntava para a gente assim: “Eu estou fazendo direitinho?”. Assim, ele foi o tempo inteiro. Era um brizolista, um pedetista, mas também um cara de carreira dentro do ministério, dentro da máquina do Estado. Apesar de trabalhar no ministério de uma ditadura, ele tinha essa franca raiz ideológica.

*Claudia Bonan: Quando o ministro da Saúde Waldyr Arcoverde conheceu a proposta do Paism, não quis dar nenhum “pitaco”, não tentou censurar?*

Nada, nada! Ele tinha muita confiança no Mozart. Mozart foi extremamente cuidadoso e, ao mesmo tempo, fiel ao que a gente propunha. Um hábil negociador. Negociou com a Igreja, rejeitou interferências do grupo do Aguinaga e da Bemfam. Houve tentativa de interferência do próprio governo, do ministro da Justiça Ibrahim Abi-Akel, que era favorável ao controle demográfico. Abi-Ackel começou a soltar umas gracinhas sobre a origem da pobreza no Brasil e atribuía ao nascimento exagerado. O Mozart teve uma habilidade, um cuidado e uma presença em conduzir a negociação... Se olhar hoje a discussão do Paism, parecia até que a gente estava em um governo democrático, tal era a lisura do processo. Isso não significa que todo o resto estava garantido, mas naquele lócus ali o diálogo foi muito, muito preservado mesmo!

*Claudia Bonan: E o Waldyr Arcoverde?*

Nós tivemos que treinar o ministro para falar na CPI e preparamos umas transparências para ele apresentar. Ele tinha aquele jeito de quem está pensando muito, aquele jeito de “espera aí, filhinha, deixa eu entender direito” [risos]. Coitado, o assunto não tinha nada a ver com a área dele. A apresentação dele não foi ruim. Foi sem entusiasmo, ele não tinha o vigor do Mozart. Eu queria que o Mozart apresentasse pelo ministério, mas tinha que ser o ministro. Eu e o Mozart ficamos atrás dele, para dar cola na hora das perguntas [risos], porque ele era um depoente da CPI, e, depois que ele falava, as pessoas perguntavam. A gente ficava ali atrás, passando bilhetinho para ele, dando apoio. Mas deu certo.

*Claudia Bonan: Como foi a reação à proposta do Paism na CPI e no governo?*

Pois é, a CPI acolheu muito bem, e no governo não houve reação, nenhuma tentativa de vetar, seja porque não compreenderam o potencial de mudanças da política, seja porque não acreditavam que aquilo prosperasse, seja porque tinham que dar alguma resposta à população, e, afinal, essa foi a resposta do ministério.

*Claudia Bonan: Sintetiza para a gente as principais inovações do Paism.*

A primeira coisa inovadora foi o Estado marcar presença e posição no tema do planejamento familiar, porque antes era omisso. Isso interessava ao governo Figueiredo. Como fazer isso é que era o pulo do gato, e a gente conseguiu dar. O Paism não era só o planejamento familiar, era um conjunto de ações envolvendo a saúde da mulher. Nessa política, o planejamento familiar deveria ser pautado na autonomia reprodutiva e na supressão de riscos à saúde das mulheres; deveria incluir ações de promoção, prevenção, educação, informação e assistência integral à saúde.

*Andreza Rodrigues: E quais foram as inovações em termos de modelos de políticas de saúde?*

Havia uma crítica grande do movimento da reforma sanitária à verticalização dos programas de saúde, que eram extremamente ineficientes, centralizadores. Eu dei o exemplo de como se planejavam as ações de saúde para um município do interior do país, uma centralização absoluta, né? Sempre defendi que o Paism tivesse um caráter mais de política do que de programa, porque um programa caminha vertical, em bloco, para o município ou o estado. Em vez de programa vertical, optamos por trabalhar com “bases para uma ação programática”, ou seja, o Paism não deveria ser uma norma vertical, imperativa, ele deveria ser um conjunto de diretrizes orientadoras. Nesse espírito, o denominamos Programa de Assistência Integral à Saúde da Mulher: bases para uma ação programática.

*Andreza Rodrigues: E quais foram as principais inovações conceituais?*

A integralidade. Jairnilson Paim atribuiu a nós a invenção desse conceito. A gente começa a formular essa vastidão, que é o conceito de integralidade, procurando olhar em todas as suas dimensões, da formulação das políticas às práticas assistenciais. Entra aí também a dimensão feminista, que é a transformação da saúde como um lugar de formação e fortalecimento da cidadania das mulheres. O lugar do respeito à subjetividade das mulheres, da não violência institucional, da autonomia, dos direitos. Nós trouxemos com o Paism um tema crítico, que precisamos ressuscitar, que é a medicalização do corpo feminino. Lembremos que a medicalização é tema caro ao movimento da reforma sanitária.

*Claudia Bonan: Depois de lançado, como começou a implementação do Paism, nos anos 1980, quando nem existia ainda o SUS?*

Levamos o Paism para as Ações Integradas de Saúde, um projeto interministerial que envolvia o Ministério da Saúde, o Instituto Nacional de Assistência Médica da Previdência Social (Inamps) e o Ministério da Educação. Hésio Cordeiro, um sanitarista de esquerda, estava na presidência do Inamps, e, na equipe técnica, havia outras feministas e sanitaristas. Com elas criamos a portaria n.3.360/1986 do Inamps, que espelha as bases de ação programática da saúde integral das mulheres. A gente já começava a trabalhar também com os estados. Os estados mais receptivos foram aqueles que tinham conselhos de direitos das mulheres.

*Andreza Rodrigues: E os movimentos sociais deram suporte?*

Entre a formulação do Paism até o seu lançamento, começou a haver uma revisão das feministas, ainda que incipiente, de alguns grupos. As feministas que eram da saúde aderiram mais rapidamente, entenderam que o Paism era também uma formulação que convergia com as angústias delas, de todas nós. No Rio, tinha o Brasil Mulher, o grupo da Fernanda Carneiro, o mandato da Lucia Arruda. Esses grupos reconheceram que o Paism representava uma inovação. Com o pé atrás, claro, porque era um governo não confiável, mas sabiam que aquilo ali era uma ilha, um projeto que representava uma possibilidade de mudança. Começa a ter mais adesões quando a gente começa a contratar os grupos para nos ajudar a formar profissionais e fazer material educativo, porque aí elas conheceram melhor a proposta. Tinha Estela Aquino, Santinha, Mariska, Martha Zanetti e outras que começaram a participar da história das práticas educativas.

*Andreza Rodrigues: Como foram as primeiras experiências de implantação nos estados e municípios?*

Eu fui para Goiás, em 1984, a convite do secretário de Saúde e com apoio do Mozart, para botar o programa para andar, para fazer uma vitrine, lugar de formação. A gente treinava médicos de dia e de noite, montava referência-contrarreferência, formava gente para fazer prática educativa com as mulheres na rede do estado e municípios. Criamos em Goiás um serviço de reversão de laqueadura, porque era um dos estados que mais tinha laqueadura tubária.

Vinha gente do Brasil inteiro ver o que a gente estava fazendo. Maria José Araújo, Marina Réa, Mariska Ribeiro, várias pessoas foram ajudar, e aí começamos a produzir material, tanto para o ministério como específico para Goiás. Aí tem anedotas, né? Eu mandei imprimir o “Nosso corpo nos pertence”, aquela série de cartilhas da Fundação Carlos Chagas, para prática educativa e formação de profissionais. O material chega numa caixa na porta da Secretaria de Saúde, e os rapazes do almoxarifado viram aquilo como um material de pornografia, e aí foi aquela confusão! O secretário me chama e fala: “Doutora, eu respeito muito a senhora, mas esse material não é científico! Veja aqui, ‘nossa vagina é bonita’!”. Então, eu perguntei: “O senhor não acha?” [risos]. Era nesse nível. Mas, anedotas à parte, expliquei que era um material muito sério, educativo, que lamentava que tivesse vazado assim. Ele acabou liberando, e a gente continuou usando. Aconteceu uma situação muito similar no ministério, quando voltei para lá. As tais cartilhas se espalharam lá. Eu fui chamada num tribunal, minha cabeça foi para os leões. O Mozart já tinha saído de cena, e eu deixei a coordenação outra vez. A área que se chamava Divisão de Saúde da Mulher e da Criança, porque a gente tinha lutado por isso, caiu na mão da Igreja, e, de novo, retornou a visão materno-infantil. Nesse contexto, a Igreja já tinha dominado o cenário e se aliado à Federação Brasileira das Associações de Ginecologia e Obstetrícia (Febrasgo) para tirar as feministas do cenário.

Do final dos anos 1980 e durante os 1990, com a presença da Igreja e da Febrasgo, tentam vetar toda a participação e presença feminista no ministério, na condução da política das mulheres. Quando Fernando Henrique assume, Ruth Cardoso queria que eu voltasse, mas não foi possível. A Febrasgo fechou questão: feminista não! Só depois, nos governos Lula, houve novo entendimento com os movimentos sociais, a reforma sanitária, as feministas...

*Andreza Rodrigues: Acho muito inspirador ouvir a história de como foi tecida a ideia e o texto do Paism. Eu tento vislumbrar como foi fazer com que esse programa conseguisse alcançar diferentes territórios e diferentes trabalhadores para que eles concretizassem essas ações...*

A ideia dessa política foi muito revolucionária, porque é uma mudança profunda de valores. É como é o feminismo. O feminismo é revolucionário, nesse sentido de desmontar estruturas de cultura do patriarcado. Nós estivemos longe de consolidar o que desejamos com o Paism. Longe! Muito distante! Mas o que fizemos, plantou muita inquietação, tanto é que estamos com 40 anos e o Paism ainda é referência.

A Política Nacional de Atenção Integral à Saúde da Mulher, de 2004, é espelhada no Paism. Ela aperfeiçoa, inclui mais grupos, tem mais complexidade temática, mas a base ético-política é a mesma: a integralidade, a autonomia, a cidadania das mulheres. Quando surge o Paism, o conceito de “gênero” nem tinha sido formulado ainda. O radicalismo possível naquele contexto histórico, orientado pelo feminismo e articulado com a reforma sanitária, dá sua tinta revolucionária. Precisamos retomar o pensamento crítico e a radicalidade das perguntas sobre saúde, autonomia, riscos, medicalização etc., que envolveram a criação do Paism, para caminhadas futuras rumo à construção da saúde integral para todas as mulheres.

*Claudia Bonan: Ana, agradecemos muito a entrevista tão rica! Essa é uma história do nosso presente, ainda estamos na luta pelos princípios e diretrizes do Paism. Então, escutar essa história na voz de uma testemunha e protagonista como você nos faz ver com mais clareza o ponto em que nos encontramos e os caminhos que temos à frente, e instrui as escolhas que devemos fazer em direção ao direito pleno e irrestrito de todas as mulheres a seus corpos, sua saúde e sua cidadania.*

## NOTAS

1 A Sociedade Civil Bem-estar Familiar no Brasil, conhecida como Bemfam, foi a primeira entidade privada de “planejamento familiar” criada no país, em 1965, e declarada organização de utilidade pública federal em 1971.

2 O Centro de Pesquisa e Atenção Integrada à Mulher e à Criança (CPAIMC) foi outra importante entidade privada de “planejamento familiar” atuante nas décadas de 1970 e 1980, encerrando suas atividades no início da década de 1990.
